# Objectively Measured Physical Activity, Sedentary Behavior and Functional Performance before and after Lower Limb Joint Arthroplasty: A Systematic Review with Meta-Analysis

**DOI:** 10.3390/jcm10245885

**Published:** 2021-12-15

**Authors:** Matic Sašek, Žiga Kozinc, Stefan Löfler, Christian Hofer, Nejc Šarabon

**Affiliations:** 1 Faculty of Health Sciences, University of Primorska, Polje 42, SI-6310 Izola, Slovenia; matic.sasek@fvz.upr.si (M.S.); ziga.kozinc@fvz.upr.si (Ž.K.); 2 InnoRenew CoE, Livade 6, SI-6310 Izola, Slovenia; 3 Andrej Marušič Institute, University of Primorska, Muzejski trg 2, SI-6000 Koper, Slovenia; 4 Physiko- & Rheumatherapie, Institute for Physical Medicine and Rehabilitation, 3100 St. Pölten, Austria; Stefan.Loefler@rehabilitation.lbg.ac.at; 5 Centre of Active Ageing, Competence Centre for Health, Prevention and Active Ageing, 3100 St. Pölten, Austria; 6 Ludwig Boltzmann Institute for Rehabilitation Research, Neugebäudeplatz 1, 3100 St. Pölten, Austria; Christian.Hofer@rehabilitation.lbg.ac.at; 7 Laboratory for Motor Control and Motor Behavior, S2P, Science to Practice, Ltd., Tehnološki Park 19, SI-1000 Ljubljana, Slovenia

**Keywords:** surgery, sitting, lower limb, rehabilitation, orthopedics, replacement

## Abstract

Patients after joint arthroplasty tend to be less physically active; however, studies measuring objective physical activity (PA) and sedentary behavior (SB) in these patients provide conflicting results. The aim of this meta-analysis was to assess objectively measured PA, SB and performance at periods up to and greater than 12 months after lower limb arthroplasty. Two electronic databases (PubMed and Medline) were searched to identify prospective and cross-sectional studies from 1 January 2000 to 31 December 2020. Studies including objectively measured SB, PA or specific performance tests in patients with knee or hip arthroplasty, were included in the analyses both pre- and post-operatively. The risk of bias was assessed using the Scottish Intercollegiate Guidelines Network (SIGN). After identification and exclusion, 35 studies were included. The data were analyzed using the inverse variance method with the random effects model and expressed as standardized mean difference and corresponding 95% confidence intervals. In total, we assessed 1943 subjects with a mean age of 64.9 (±5.85). Less than 3 months post-operative, studies showed no differences in PA, SB and performance. At 3 months post-operation, there was a significant increase in the 6 min walk test (6MWT) (SMD 0.65; CI: 0.48, 0.82). After 6 months, changes in moderate to vigorous physical activity (MVPA) (SMD 0.33; CI: 0.20, 0.46) and the number of steps (SMD 0.45; CI: 0.34, 0.54) with a large decrease in the timed-up-and-go test (SMD −0.61; CI: −0.94, −0.28) and increase in the 6MWT (SMD 0.62; CI: 0.26–0.98) were observed. Finally, a large increase in MVPA (SMD 0.70; CI: 0.53–0.87) and a moderate increase in step count (SMD 0.52; CI: 0.36, 0.69) were observed after 12 months. The comparison between patients and healthy individuals pre-operatively showed a very large difference in the number of steps (SMD −1.02; CI: −1.42, −0.62), but not at 12 months (SMD −0.75; −1.89, 0.38). Three to six months after knee or hip arthroplasty, functional performance already exceeded pre-operative levels, yet PA levels from this time period remained the same. Although PA and functional performance seemed to fully restore and exceed the pre-operation levels at six to nine months, SB did not. Moreover, PA remained lower compared to healthy individuals even longer than twelve months post-operation. Novel rehabilitation protocols and studies should focus on the effects of long-term behavioral changes (increasing PA and reducing SB) as soon as functional performance is restored.

## 1. Introduction

Osteoarthritis (OA) is the most prevalent degenerative disease of the musculoskeletal system [1]. In many cases, especially in older adults, it causes joint pain and limits functional ability. When the joints of the lower limbs are affected, weight-bearing activities, such as walking or kneeling, can be severely impaired [2]. Because severe OA of the lower limb joints drastically affects quality of life, hip and knee arthroplasty are being considered as viable treatment options when conservative treatment does not lead to the relief of symptoms. The increasing prevalence of OA associated with ageing of the population is reflected in a proportionally higher number of total joint arthroplasty procedures. Consequently, total hip and knee arthroplasties are among the most common elective surgical procedures worldwide [3,4]. The commonly expected outcomes of surgery are pain relief, increased mobility, function and higher quality of life, which are associated with increased physical activity (PA) levels and sport participation [5,6,7].

Sedentary behavior (SB) is one of the major risk factors for developing the chronic non-communicable disease. Together, these diseases are estimated to cause 71 percent of all deaths worldwide [8]. Therefore, reducing SB is useful for improving longevity, long-term health and well-being. It is even better to replace SB with PA, which is highly effective in preventing and treating diseases [9,10]. According to the latest guidelines, 150–300 min of moderate to vigorous intensity or 75–150 min of vigorous intensity PA per week is considered sufficient to maintain health and well-being [11]. Because of its physiological benefits, PA is a part of rehabilitation for a variety of disabilities, including osteoarthritis and joint arthroplasty. The dose–response relationship between levels of PA and health in middle-aged and older adults is strong [12]. The positive effects of physical activity on health are well established in practice and have a strong theoretical background. Finally, the cost-effectiveness of physical activity from the perspective of an individual’s health and social and economic well-being is undisputed [13,14].

Over the last two decades, the development of high-technology measurement tools has enabled the quantification of PA and SB levels. Compared to questionnaires, new-generation accelerometers provide a more reliable and objective insight into an individual’s physical activity profile [15]. Many systematic reviews and meta-analyses have examined objectively measured and self-reported PA levels or sport participation in patients after lower limb arthroplasty [7,16,17,18,19]. Notably, physical function is shown to recover to approximately 80% of that of controls [20]. To our knowledge, only one systematic review considered objectively measured SB after TKA and provided heterogenous results. The authors suggested that knowing SB post-operative trajectories in detail would help in tailoring targeted interventions [16]. Although total joint arthroplasty increases functional capacity and relieves OA-related pain, it remains unclear whether and to what extent patients change their objectively measured PA, SB and functional performance post-operation [17,21].

For this purpose, the primary aim of this paper was to review the studies that prospectively investigated objectively measured PA, SB and functional performance after lower limb arthroplasty for up to 12 months. The secondary aim of the study was to pre- and post-operatively compare the OA patients with healthy peers. We hypothesized that (a) patient groups will exhibit lower PA, higher SB and lower functional performance compared to healthy peers and (b) these outcomes will improve throughout the first 12 months following the operation. While some systematic reviews regarding this topic already exist, the number of studies reporting objectively measured PA and SB have been increasing in the last years. Numerous studies published enabled us to provide objectively measured PA and SB change trajectories. By including functional performance changes, a coherent interpretation off all three determinants together could be of high value for future clinical research and rehabilitation interventions of patients after lower limb arthroplasty in accordance with the Global Physical Activity Action Plan [22]. The review was performed according to the PRISMA 2020 statement for reporting systematic reviews [23].

## 2. Materials and Methods

### 2.1. Search Strategy

The study protocol was conducted in accordance with the Cochrane Collaboration guidelines [24]. Two electronic databases, including PubMed and Medline, were searched from 1 January 2000 to 31 December 2020. The literature search was conducted using the keywords and terms used in the search strategy, including: “physical*” “active*” OR “sedentary” OR “sitting” AND “endoprosthesis” OR “replacement” OR “arthroplasty” OR “arthrosis” OR “arthritis” OR “osteoarthrosis” OR “osteoarthritis” AND “knee” OR “hip” OR “ankle”. One author searched the databases for the consistency of search hits. In addition, the reference lists of all included articles were searched for relevant studies that were not covered by the constructed search strategy.

### 2.2. Eligibility Criteria

Studies were included if they met the following inclusion criteria: (a) the study design was longitudinal or cross-sectional and included pre-operative and at least 3 months of post-operative data, (b) the study reported on objectively measured PA or SB or measured functional performance by using the 6 Minute Walk Test (6MWT) or Time Up and Go (TUG) and (c) included adult participants with a diagnosis of knee or hip OA who underwent primary or recurrent unilateral or bilateral total or complementary joint arthroplasty. For PA, only objective methods (uni-, bi- or triaxial accelerometers or pedometers) were considered. For SB, only accelerometers were accepted as an objective method of assessment. Reviews were not included in the systematic review, but the reference list of reviews found was examined, and all studies that met the inclusion criteria were included. Studies were excluded from the review if they (a) measured PA or SB using subjective questionnaires or performance level with other tests or (b) the results were obtained only for a single timeframe (i.e., only 12 months post-operation).

### 2.3. Study Selection and Quality Assessment 

The abstracts and titles of the articles were screened for eligibility by two reviewers. The full text was than obtained and reviewed by two reviewers for articles with limited eligibility and potentially eligible articles. The articles whose eligibility criteria were accepted by both reviewers after the full text review were included in the review. Any ambiguities or conflicts were resolved through discussion. To detect bias and assess methodological quality, the Scottish Intercollegiate Guidelines Network (SIGN) methodology for cohort studies was used (Table S1). One author reviewed the quality of the studies and graded them as high (++; major of criteria met), acceptable (+; most criteria met) or low (0; most criteria not met) [25].

### 2.4. Data Items

For data extraction, a prepared data sheet was used by one reviewer and checked for accuracy and consistency by a second reviewer. For all included articles, the first author, year of publication, study design, type and location of surgical procedure (knee arthroplasty (KA) or hip arthroplasty (HA) and type of procedure), participant characteristics (age, body mass index and gender distribution), type of accelerometers and outcomes were extracted and summarized in tables in sequential order. For outcomes PA, SB and functional performance, data were collected in chronological order at one pre-operative and four post-operative time periods. Means, standard deviations and sample sizes were extracted from included studies. For articles in which results were reported in medians, interquartile ranges and confidence intervals, means and standard deviations were calculated using the methods of Wan [26] and Moher [27].

### 2.5. Data Analysis

Meta-analyses were performed using Review Manager software (version 5.3, Cochrane Collaboration, London, UK). The inverse variance method with the random effects model was used. Differences (between patients and controls or between time points) were expressed as standardized mean differences (SMDs) and corresponding 95% confidence intervals (Cis). When possible, mean differences were also calculated in units of measurement. Statistical heterogeneity between studies was assessed by calculating the I^2^ statistic. According to the Cochrane guidelines, I^2^ statistics of 0% to 40% may not be important, whereas 30% to 60% represent moderate heterogeneity, 50% to 90% represent substantial heterogeneity and 75% to 100% represent substantial heterogeneity. Sensitivity analysis was performed by eliminating studies one-by-one and checking whether the statistical significance of the pooled effect was affected. 

## 3. Results

**Figure 1:**
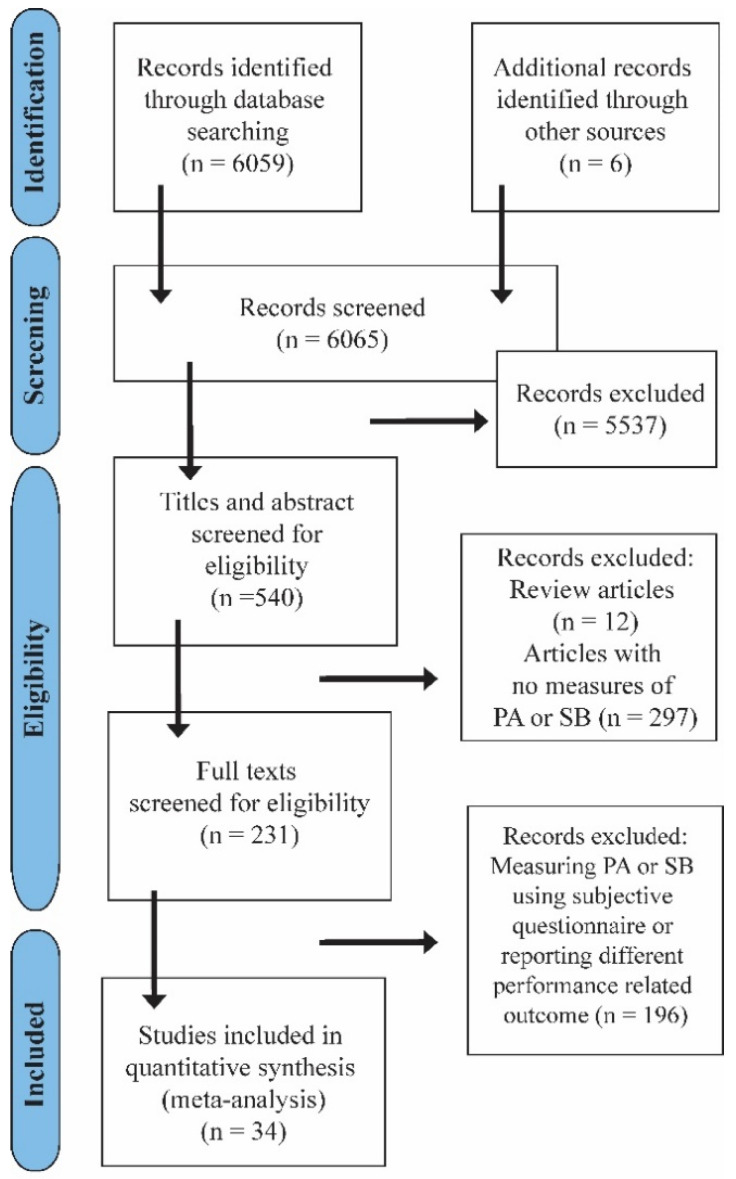
Summary of study search protocol. PA—physical activity; SB—sedentary behavior.

The initial search yielded 6059 results in the databases. Thirty-four studies met the inclusion criteria and were included in the review (Table 1). The number of steps was reported for 919 subjects (689 female and 230 male) with a mean age of 63.7 (±7.5) and 29.1 (3.7) BMI; MVPA for 358 subjects (266 female and 92 male) with a mean age of 67.9 (4.9) and 27.1 (2.7) BMI; SB for 230 subjects with a mean age of 66.5 (3.6) and 29.7 (2.9) BMI; 6MWT for a mean age of 63.7 (3.2) and 30 (2.4); and TUG for 192 subjects with average age 65.1 (2.9) and 28.6 (2.9) BMI. The flowchart of the selection process according to the PRISMA statement [27] is shown in Figure 1. Twenty-seven studies were classified as acceptable (+), seven studies were classified as high quality (++) and only one study was classified as low quality (0) (Table S1). 

### 3.1. Comparison of PA between Patients and Healthy Individuals Pre- and Post-Operation

**Figure 2:**
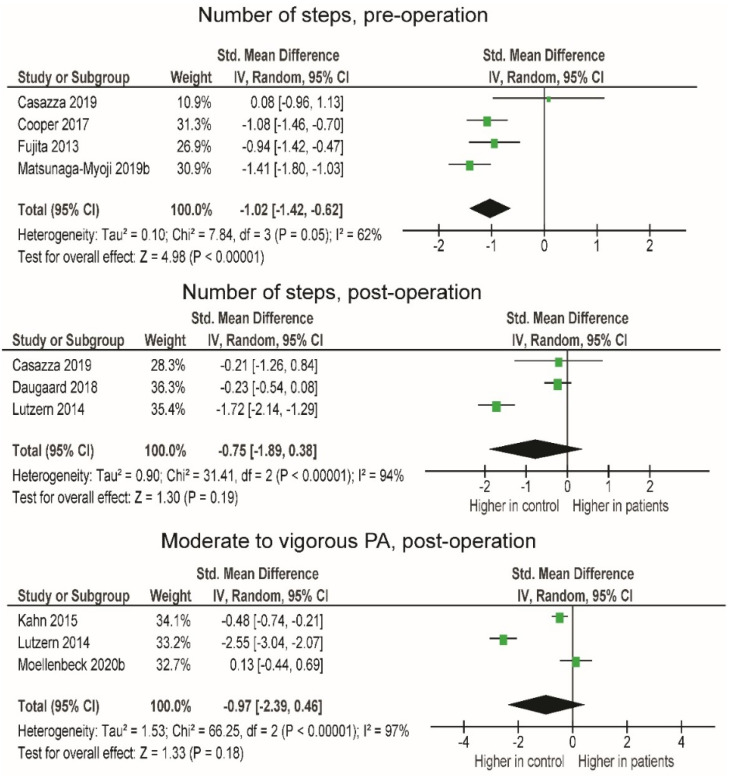
Number of steps and moderate to vigorous physical activity in patients and controls, pre-operation and 12 months post-operation. CI—confidence interval; I^2^—heterogeneity statistics.

In the first set of analyses (Table 2), we compared patients with matched control groups pre-operation. There was a very large difference in the number of steps (Figure 2; SMD = −1.02; *p* < 0.001), with the patients performing, on average, 2892.2 less steps per day compared to the control group. In the second set of analyses, we compared patients with matched control groups 12 or more months post-operation. The amount of moderate to vigorous physical activity (MVPA) (SMD = −0.97), as well as the number of steps (SMD = −0.75), tended to be lower in patients. However, despite large effect sizes, the differences were not statistically significant (*p* = 0.180–0.190), likely due to the large heterogeneity between studies (I^2^ = 94–97%).

### 3.2. Comparison of PA and SB in Patients Pre- and Post-Operation

**Figure 3:**
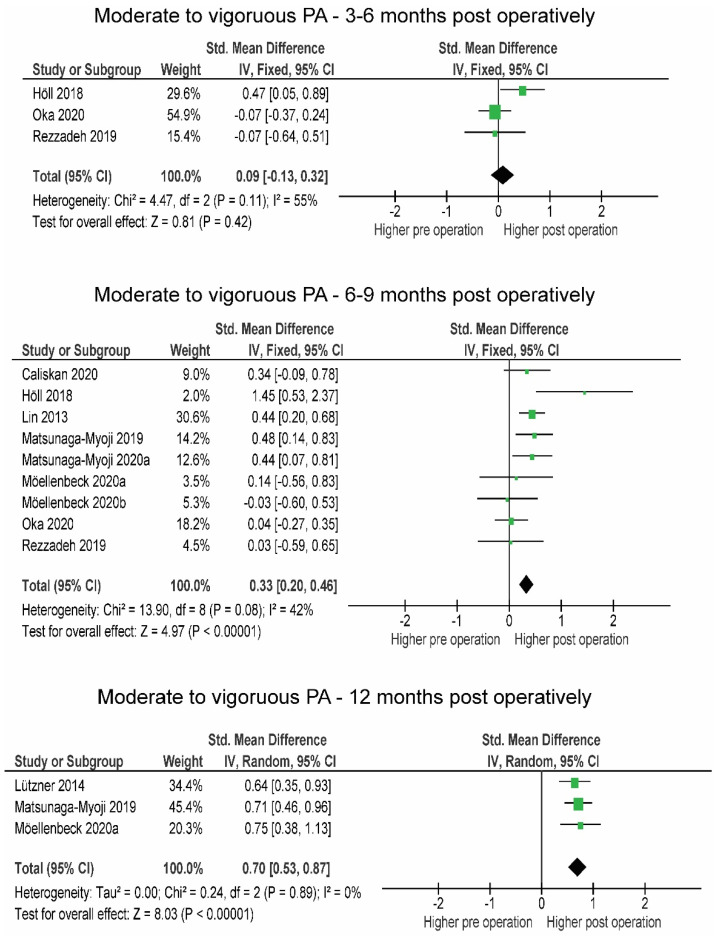
Changes in moderate to vigorous physical activity over the time post-operation. CI—confidence interval; I^2^—heterogeneity statistics.

Between 3 and 6 months post-operation, MVPA remained at the pre-operative level (SMD = 0.12, *p* = 0.530), increased between 6 and 9 months (SMD = 0.33, *p* < 0.001) and remained greater more than 12 months post-operation (SMD = 0.70, *p* < 0.001) (Figure 3).

Light-intensity physical activity also tended to increase (SMD = 0.14; *p* = 0.160), while SB was similar pre-operation and between 6 and 9 months post-operation (SMD = −0.00, *p* = 0.790).

**Figure 4:**
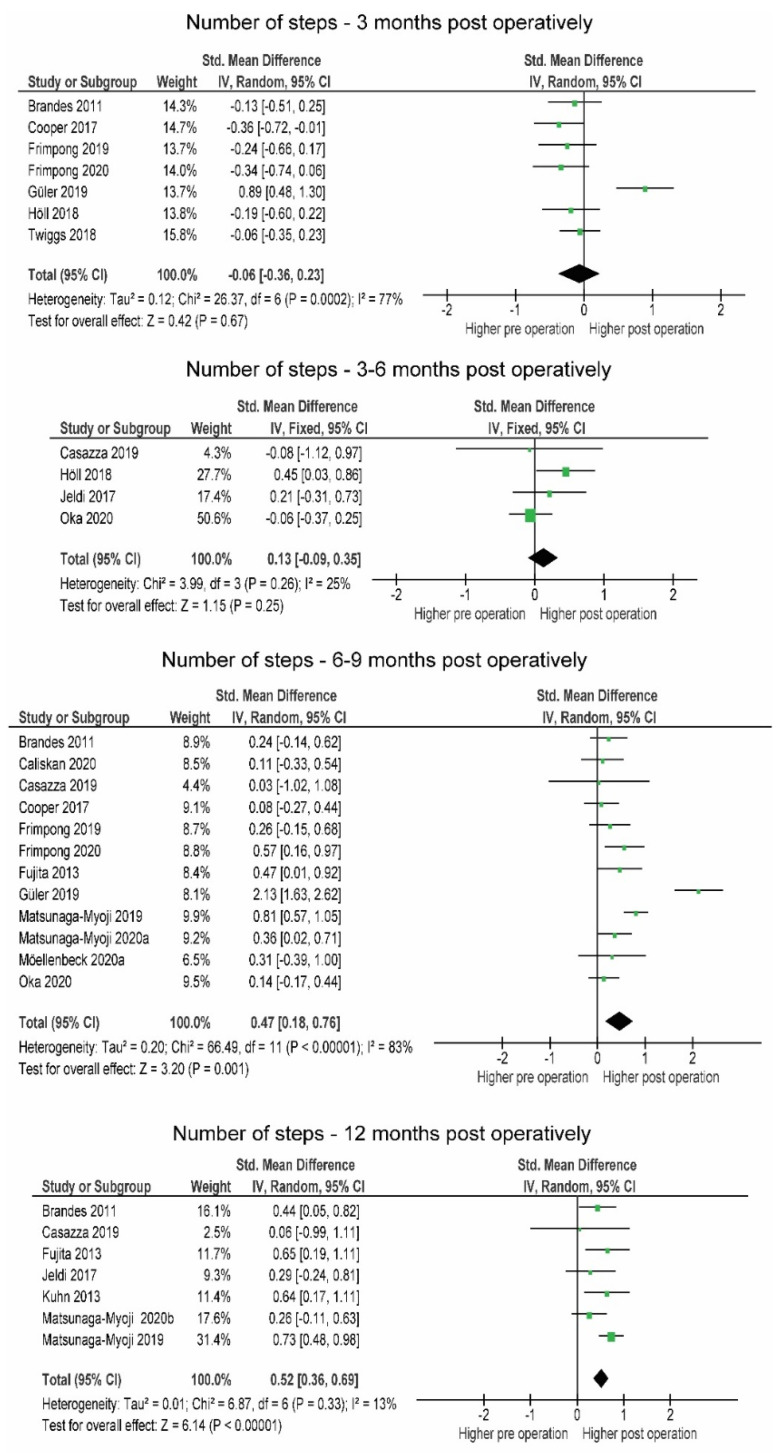
Changes in number of steps over the time post-operation. CI—confidence interval; I^2^—heterogeneity statistics.

**Figure 5:**
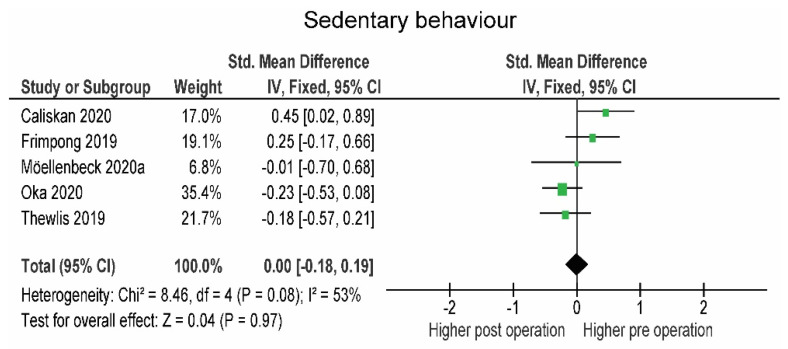
Changes in sedentary behavior 6 to 9 months post-operation. CI—confidence interval; I^2^—heterogeneity statistics.

The number of steps (−196.7; SMD = −0.06) tended to be lower within 3 months post-operation, but the differences were not statistically significant (*p* = 0.670). Three to six months post-operation, patients slightly but not significantly (*p* = 0.390) increased the number of steps (+373.8, SMD = 0.15). At 6 months, patients significantly exceeded the pre-operative level (+1064.1, SMD = 0.45, *p* < 0.001), which was exceeded 12 months post-operation (+1425.3, SMD = 0.52, *p* < 0.001) (Figure 4). Results regarding sedentary behavior were inconsistent (I^2^ = 53 %) and amounted to exactly zero overall effect (SMD = 0.00) (Figure 5).

### 3.3. Comparison of Functional Performance in Patients Pre- and Post-Operation 

**Figure 6:**
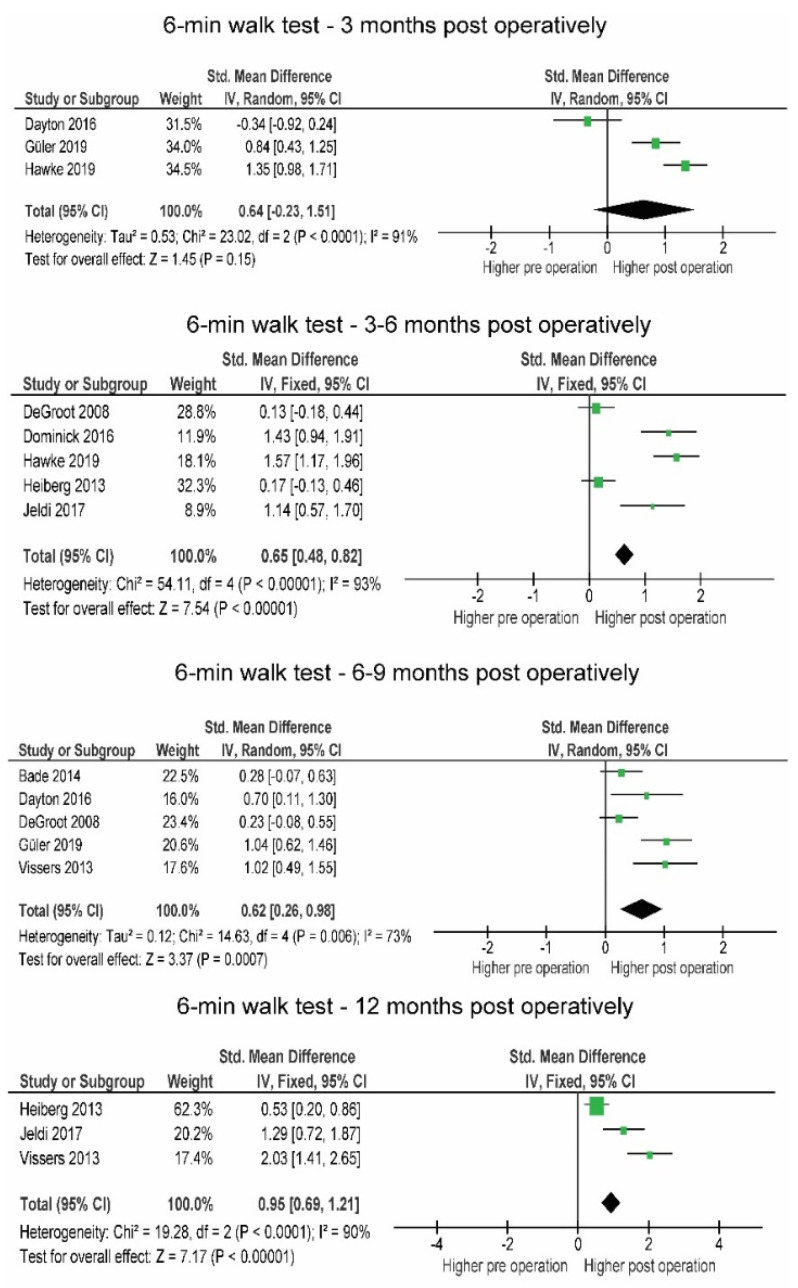
Changes in 6 min walk test over the post-operative time period. CI—confidence interval; I^2^—heterogeneity statistics.

In the first 3 months post-operation, the patients tended to improve their 6MWT (+59.9 m; SMD = 0.64) compared to the pre-operative level, but not enough to confirm this statistically (*p* = 0.15) (Figure 6). At this time, the results of the TUG test still indicated a slight, statistically non-significant impairment in function (+1.58 s, SMD = 0.27, *p* = 0.770).

**Figure 7:**
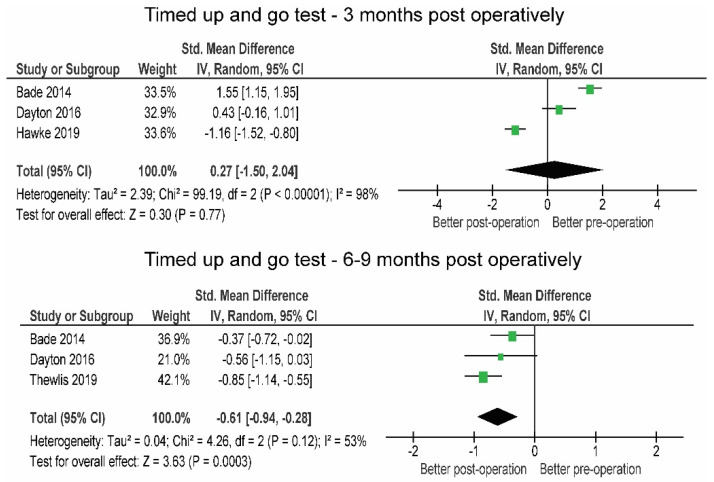
Changes in 6 min walk test over the post-operative time period.

Three months post-operation, the results of the 6MWT (+90.2 m; SMD = 0.87) were largely and statistically significantly (*p* = 0.008) improved compared to pre-operation and remained improved between 6 and 9 months (+71.84 m; SMD = 0.62; *p* < 0.001). The TUG test was also largely and significantly (−1.91 s; SMD = −0.61; *p* < 0.001) improved between 6 and 9 months compared to pre-operative levels (Figure 7).

Table 3 summarizes the obtained evidence across the different time intervals. 

### 3.4. Sensitivity Analyis

According to the sensitivity analyses for all outcomes at all time points, no single studies were identified that would change the statistical significance of the pooled effect size. 

## 4. Discussion

The primary aim of this systematic review was to determine whether total arthroplasty is associated with changes in objectively measured PA, SB and functional performance at different post-operation follow-up periods to examine the progression of recovery after lower limb arthroplasty.

### 4.1. Comparison of PA between Patients and Healthy Subjects Pre- and 12 Months Post-Operation

Patients were less physically active than healthy peers both pre- and 12 months post-operation (Figure 2). Specifically, a lower number of steps was observed in patients pre-operation. Studies by Fujita et al. [39] and Matsunaga-Myoji et al. [51] suggest that the level of light PA is also lower in OA patients awaiting surgery than in healthy individuals. In addition, Moellenbeck et al. [55] and Matsunaga-Myoji et al. [51] found that healthy subjects spend more time doing MVPA than OA patients. The observed trend could be a logical consequence of OA symptoms preventing weight-bearing PA and increasing SB. It seems that the discrepancy in MVPA and performance between controls and patients is still evident 12 months or more after total joint arthroplasty [46,62]. Although OA pain has been shown to be decrease post-operation, patients do not reach the performance and activity levels of healthy individuals even at 12 months or more post-operation.

### 4.2. Comparison in PA, SB and Performance of Patients Pre- and Post-Operation

Prospective observations of patients are more numerous in the literature than comparisons with healthy controls. In this section, pre-operative PA and SB levels as well as functional performance at different time intervals were compared to post-operative levels.

#### 4.2.1. Post-Operative Changes in PA

In the first three months post-operation, the number of steps remained the same as those observed pre-operation. When the study by Güler et al. [40] is excluded from the analysis, significantly more steps are taken pre-operation (Figure 2). Nevertheless, it may not be reliable to draw conclusions about the general level of PA based on the number of steps, as steps are only a partial survey of the frequency of physical activity. Intensity and duration need to be recorded to more accurately assess the level of PA. In the prospective study, Frimpong et al. [37] observed that within the first three months post-operation, patients spent 27 min per day less engaging in moderate intensity PA than pre-operation and maintained the level of SB. Similarly, Höll et al. [43] observed that about 300 fewer steps per day were taken shortly after surgery at light-intensity PA and also noted a slight increase in MVPA. When making comparisons between studies, we must consider that the measurements were taken at different post-operative times: 2 and 10 weeks. Therefore, those who measured PA earlier likely noted greater impairment due to higher acute inflammatory processes limiting overall activity and function. Intensive physiotherapy during this period must also be taken into account as it may affect the patient’s overall PA levels. It is very likely that post-operative limitations such as pain and protection of the affected limb affect PA within the first three months post-operation [63,64].

Four studies were included in the analysis of the number of steps and three studies in the analysis of MVPA between 3 and 6 months post-operation (Figure 1 and Figure 2). Very little difference was observed in the number of steps (+333 steps/day), while no difference was observed in MVPA. Only two studies [56,58] observed SB, and in both, patients already reached but did not exceed pre-operative levels. Light-intensity physical activity remained the same, as noted in the studies by Thewlis et al. [58] and Oka et al. [56] (from 329 pre- to 316 min/day post- and from 239 pre- to 232 min/day post-, respectively).

At 6 to 9 months post-operation, MVPA and the number of steps were above pre-operative levels. These observations are supported by previous findings by Mills et al. [17], who only found a minimal increase in MVPA 6 months post-operation. The slight discrepancy in MVPA between studies could be due to differences in the interpretation of variables describing PA and differences in methodology and the number of studies included in the analyses. The main determinants in the study by Mills et al. [17] were time spent in locomotion and time spent active, while we used MVPA and SB as PA determinants.

The results of the prospective studies with a follow-up period of 12 months or longer showed high homogeneity and a trend toward increased number of steps (Figure 4). Three studies in which MVPA was observed provided a large effect size, indicating a long-term post-operative increase in PA. The results of our study showed that patients fully regained and exceeded their pre-operative PA level after 6 and 12 months. Similar results were previously reported in two systematic reviews by Arnolad et al. [65] and Mills et al. [17], where improvements in PA over time, especially after 12 or more months, were evident.

#### 4.2.2. Post-Operative Changes in SB

Three studies compared SB less than 3 months [37,58] and from 3 to 6 months [56,58] post-operation. The time trajectory of SB is clearly seen from the study by Thewlis et al. [58], in which SB increases from pre-operation to 2 weeks post-operation for 120 min/day and decreases back to pre-operative levels after 12 weeks (620 min/day to 624 min/day). A similar trend was observed by Oka et al. [56], while Frimpong et al. [37] reported that SB was already recovered after 6 weeks post-operation.

Most of the SB data were provided from 6 to 9 months post-operation. On average, the values of SB remained constant and did not significantly decrease 6 months post-operation, similar to the study by Frimpong et al. [38] in which only a small decrease in sitting time was observed at 6 months (~5%). In general, SB is not significantly reduced in a time period from 6 to 9 months, but in the study by Möellenbeck et al. [54], sedentary activities longer than 60 min were significantly reduced in older adult patients. As long-term objectively measured SB data are lacking and studies report divergent SB variables, future research should primarily focus on methodological quality and long-term outcomes.

#### 4.2.3. Post-Operative Changes in Functional Performance 

In the first three months post-operation, 6MWT and TUG increased slightly but not significantly. It is logical that patients regain pre-operative functional performance levels more related to general locomotion ability, such as walking at a steady pace, sooner after arthroplasty than more complex tasks such as explosive strength and agility. The subsequent restoration of TUG could be due to the fact that surgery-related swelling and pain do not enable one to perform at peak functional capacity since strength deficits of lower limbs might be present [66].

At 3 to 6 months, the 6MWT was significantly higher than the pre-operation, suggesting that the surgically induced pain might be reduced and functional capacity restored by arthroplasty [66]. It has been reported that the subjective perception of functional ability at this time period was lower than pre-operation [67], so there may be some discrepancies between actual functional ability and the subjective perception of functional ability due to post-operative anxiety and uncertainty of the patients. Our results showed that six months post-operation, patients exceed their pre-operation functional ability. Restored functional capacity allows patients to participate in sport, as Witjes et al. [68] reported that the majority of patients after lower limb arthroplasty who were previously physically active returned to high-impact sports after 26 weeks.

#### 4.2.4. Coherent Interpretation of PA, SB and Functional Performance Changes

Overall, objectively measured PA appears to increase over time, but patients are unable to reach pre-operative levels of PA until about 6 months post-operation, when PA values match and then exceed pre-operative levels. Functional performance, on the other hand, tends to increase earlier (3 months post-operation) and continues to develop up to 12 months post-operation. Although functional performance is restored at 3 months, MVPA remains at pre-operative levels. Moreover, SB remains unchanged in the period between 6 and 9 months. Since the main goals of rehabilitation after joint arthroplasty are to maximize functional performance, optimize lifestyle and promote patient independence to improve overall health [69], increasing PA and reducing SB should be incorporated and encouraged as soon as possible. The results of this study show that even if functional performance is increased, patients remain as sedentary as they were before arthroplasty. Only PA recovered, albeit with a delay, and significantly exceeded the pre-operative level at 12 months post-operation. Although arthroplasty significantly restored patients’ function, patients remained less physically active than their healthy peers 12 months post-operation.

According to Bull et al. [11], adults and older adults should spend at least 150–300 min per week in MVPA to achieve substantial health benefits of physical activity. Only in the studies by Moellenback et al. [55], Oka et al. [56] and Hylkema et al. [70] did patients achieve these recommendations 6 and 12 months post-operation. On average, functional performance and PA increased after the arthroplasty, but not enough to reach the general health guidelines. More importantly, SB remained unchanged. Therefore, novel rehabilitation protocols could solve the problem of achieving sufficient PA and reducing SB in the long term. Peter and colleagues [71] already proposed to expand the current recommendations for the rehabilitation process after lower limb arthroplasty. A behavioral approach could be applied in line with recent guidelines, which aim to limit SB and frequently interrupt it by PA to make it less harmful to overall health [11]. In addition, supervision and a motivational approach to rehabilitation after KA and HA have provided promising results [72,73,74], so the idea of a rehabilitation protocol incorporating high technology supporting a contemporary behavioral therapy approach seems reasonable preference in patients undergoing lower limb arthroplasty.

Because the study has several potential limitations, the results must be interpreted with caution. First, high heterogeneity was found in the analyses for some outcomes. This could be due to the fact that we comprehensively analyzed HA and KA, so high heterogeneity between the two subgroups could bias the results. Second, the data came from studies that used different activity monitors (bi-, uni- or triaxial accelerometers) with different reliability, validity and outcomes. Third, some authors have pointed out that measuring PA with objective data alone underestimates the realistic level of PA, so a combination of subjective and objective measurements is more appropriate when interpreting PA of patients after total joint arthroplasty [17]. Finally, we used the number of steps as a PA determinant, although it is not as well associated with general health as PA and SB [75]. We chose to do so because it has been most commonly used as a measure of PA and may help, at least in part, to explain the trajectories of PA in combination with MVPA and SB. To provide stronger evidence for practical implications, future studies examining PA and SB in patients after lower limb arthroplasty should use similar methodology and reliable instruments that provide objective data. The limitation of the current literature is that the studies either (a) compared patients and controls in single time points or (b) only tracked patients prospectively. Therefore, the progress of the patients with respect to the control groups is difficult to discern.

## 5. Conclusions

The results suggest that objectively measured PA and functional performance increase while SB remain unchanged after lower limb arthroplasty. However, an optimal lifestyle is not achieved. Functional performance soon after surgery exceeds pre-operative levels and increases over time. In the period before and more than 12 months post-operation, patients tend to have lower functional capacity and are less physically active than their healthy comparison groups. Since the main goal of rehabilitation after lower limb arthroplasty is to improve patients’ functional ability and general health, novel long-term rehabilitation approaches should be adapted to influence patients’ lifestyle and in a way that maintains or even improves patients’ overall health in the long term. 

## Figures and Tables

**Table 1:** Summary of included studies objectively measuring physical activity, sedentary behavior and performance pre and post lower limb arthroplasty.

Study	Study Design	Pre-Operative Descriptive Statistics	Type and Location of Arthroplasty	Outcome	Measurement	Main Conclusions
Bade 2014 [28]	Prospective observational study	n = 64 sex = 50% F, 50% M; age = 64.6 ± 8.5 BMI = 30.6 ± 4.8	Tricompartmental, cemented TKA with the medial parapatellar approach.	Performance: 6MWT, TUG	Pre: 1–2 weeks Post: 6 months	Acute post-operative TUG performance can be used for establishing a prognosis.
Brandes 2011 [29]	Prospective observational study	n = 53 sex = 63% F; 27% M age = 65.8 ± 5.8 BMI = 30.7 ± 4.1	Cemented and uncemented TKA.	SB and PA: tri-axial DynaPort Activity Monutor (McRoberts); Step activity monitor (OrhoCare Innovations)	Pre: 1–2 weeks Post: 2 months, 6 months, 12 months	The activity level post treatment seems to be influenced by physical activity behavior prior to surgery rather than by the treatment itself.
Caliskan 2020 [30]	Prospective observational study	n = 36 sex = 86% F; 14% M age = 67.3 ± 7.7 BMI = 33.2 ± 5.9	Primary TKA.	SB and PA: ActiCal (Philips Respironics)	Pre: 1 week Post: 6 months	No change in sedentary behavior time, increased light physical activity and moderate to vigorous activity.
Casazza 2019 [31]	Prospective control study	n = 7 sex = 5 F; 2 M age = 55.6 ± 3.5 BMI = 32.8 ± 1.5	Primary TKA.	SB and PA: Sensewear Pro 3 (BodyMedia)		Non-significant trends towards improved CDV fitness and activity levels 12 months after surgery.
Cooper 2017 [32]	Prospective control study	n = 62 sex = 58% F; 42% M age = 60.5 ± 10.3 BMI = 33.3 ± 6.7	TKA.	SB and PA: single axis accelerometer (PAL Technologies).	Pre: Post: 6 weeks, 6 months	Daily function only returns to the pre-operative level after six months.
Daugaard 2018 [33]	Cross-section study	n = 52 sex = 50% F; 50% M age = 62 ± 9.6 BMI = N/A	Primary unilateral total or unicompartmental knee replacement surgery.	SB and PA: tri-axial accelerometer X16-mini (GCdataconcepts) and AX3 (Axivity)	Post: at least 5 years	Knee OA and treatment with joint replacement hardly affect health-related general activity but affects specific behavior.
Dayton 2016 [34]	Prospective observational study	n = 23 sex = 16 F; 7 M age = 61.4 ± 8.3 BMI = 29.2 ± 5.1	Primary unilateral posterior approach THA due to OA. Between June 2010 and August 2011.	Performance: 6 MVT, TUG	Pre: 2 weeks Post: 1 month, 6 months	Performance seems to increase 6 months post-operation.
De Groot 2008 [35]	Prospective observational study	n = 44 TKA; n = 36 THA sex = TKA: 55% F, 45% M; THA: 64%F; 36% M age = TKA: 62.1 ± 9.7; THA: 61.5 ± 12.8 BMI = TKA: 26.6 ± 4.2; THA: 32.1 ± 5.3	Posterolateral approach THA. TKA procedures using computer navigation (Brainlab, Faldkirchen, Germayn) were performed.	SB and PA: Activity monitor accelerometer (AM)	Pre: 2 months Post: 3 months, 6 months	Patients did not adopt a more active lifestyle 6 months after surgery despite improvements in other aspects of physical functioning.
Dominick 2018 [36]	Prospective observational study	n = 41 sex = 68.3% F; 31.7% M age = 65.4 ± 7.9 BMI = 34.4 ± 7.7	Primary elective TKA for knee OA.	Performance: 6 MVT	Post: 1 month, 3 months	There is a complex and poorly understood relationship between thoughts, behaviors and physical impairments.
Frimpong 2019 [37]	Sub-study of prospective observational study	n = 49 sex = 90% F; 10% M age = 62.8 ± 8.6 BMI = 33.8 ± 7.1	Primary TKA.	SB and PA: GTX3 (ActiGraph)	Pre: 2 weeks Post: 6 weeks, 6 months	Objectively measured light physical activity increase and sedentary behavior decreased 6 months after TKA.
Frimpong 2020 [38]	Prospective observational study	n = 45 sex = 93% F; 7% M age = 63.8 ± 8.8 BMI = 34.6 ± 7.8	Primary TKA from August 2015 to April 2017.	SB and PA: activPAL (PAL Technologies)	Pre: 2 weeks Post: 6 weeks, 6 months	Decreasing pain and reducing functional limitation have no effect on changes in activity behavior in obese patients with knee OA.
Fujita 2013 [39]	Prospective control study	n = 38 sex = 100% F age = 60.9 ± 9.1 BMI = 23.0 ± 3.6	Primary THA for OA.	PA: Lifecoder SX pedometer (Suzuken)	Pre: 4 weeks Post: 6 months, 12 months	In the patients, all physical activity indicators improved significantly over time and reached 80–90% of those in the control group 12 months after THA.
Güler 2019 [40]	Prospective control study	n = 50 sex = 72% F; 28% M age = 57.1 ± 13 BMI = 29.2 ± 5.2	Anterolateral approach THA with uncemented prothesis between October 2014 and October 2015.	PA: Pedometer TKS1257 (BTM life)	Pre: NA Post: 6 weeks, 6 months	Patients with OA showed improved physical function and activity as early as six weeks and up to six months after THA.
Hawke 2019 [41]	Prospective observational study	n = 54 sex = 57% F; 43% M age = 67.8 ± 8 BMI = N/A	Primary total hip or knee joint replacement.	Performance: 6MWT, TUG	Post: 0 weeks, 6 weeks, 12 weeks	Walking performance increased after group-based therapy and continued to improve after group discharge.
Heiberg 2013 [42]	Prospective observational study	n = 88 sex = 58% F; 42% M age = 66 (64–68) BMI = 27 (26–27)	Primary THA from October 2008 to June 2010.	Performance: 6MWT	Pre: Post: 3 months, 12 months	After THA performance improved slowly through the first post-operative year.
Höll 2018 [43]	Prospective observational study	n = 46 sex = 54.4% F; 45.6% M age = 63.3 ± 10 BMI = 27.1 ± 4	Minimal invasive, direct anterior approach THA.	PA and SB: Step-Watch 3TM Activity Monitor (Orthocare Innovations)	Pre: 1 week Post: 6 weeks, 3 months	Objectively measured PA takes longer than 6 weeks for significant improvements.
Jeldi 2017 [44]	Prospective observational study	n = 30 sex = 70% F; 30% M age = 67 (50–82) BMI = 31 (19–43)	Uncemented an cemented posterior approach THA.	PA and SB: activPAL3 (PAL Technologies)	Pre: within 2 weeks Post: 3 months, 12 months	No change in the volume of PA 12 months post-operation.
Kahn 2015 [45]	Cross section study	n = 63; n = 60 sex = 49.2% F; 50.8%; 50% F; 50% M age = 68.4 ± 8.2; 67.3 ± 8.7 BMI = 29.2 ± 4.8; 31.1 ± 5.3	Primary TKA.	PA and SB: ActiGraph GT1M (ActiGraph)	Pre: 552.6 ± 358.9 days Post: 624.8 ± 420.6 days	No significant difference in physical activity levels between the OA and TKA group.
Ko 2013 [46]	Randomized control study	n = 32 sex = 56% F; 44% M age = 66.7 (64.3–69.2) BMI = 30.8 (27.6–34.9)	Primary TKA.	Performance: 6MWT, TUG	Post: 12 to 18 months	Controls performed significantly better in both the TUG and 6MWT.
Kuhn 2013 [47]	Prospective observational study	n = 37 sex = 68% F; 32% M age = 42.1 ± 7.2 BMI = 29 ± 5.6	Primary THA.	PA and SB: StepWatch Activity Monitor 3.0 (Cyma Corp.)	Pre: NA Post: 1.3 ± 0.2 years	Significant improvement in physical activity level and intensity was observed in patients.
Lin 2013 [48]	Prospective observational study	n = 12 sex = 100% F age = 58.2 ± 3.7 BMI = 23.4 ± 4.1	THA using Secur-Fit Plus Max stem (Stryker), Trident Acetabular Shell (Stryker) and Trident Polyethylene Bearing (Stryker) implants.	PA and SB: RT3 accelerometer (StayHealthy)	Pre: 1 month Post: 6 months	Patients did not develop a more active lifestyle, but they increased the amount of moderate and vigorous activities after surgery.
Lützner 2014 [49]	Prospective observational study	n = 97 sex = 46.4% F; 53.6% M age = 68.9 (67.2–70.6) BMI = 31.3 (30.3–32.3)	Unconstrained TKA between March 2009 and September 2011.	PA and SB: activPAL (PAL Technologies)	Pre: 1 week Post: 12 months	Moderate improvement in the total number of steps, but no change in daily walking time.
Lützner 2016 [50]	Prospective observational study	n = 221 sex = 56.6% F; 43.4% M age = 68.1 ± 9.5 BMI = 31.3 ± 4.9	Unconstrained bicondylar TKA.	PA and SB: activPAL (PAL Technologies)	Pre: 1 weekPost: 12 months	MVPA and the number of steps were significantly increased 12 months follow up.
Matsunaga-Myoji 2019 [51]	Prospective control study	n = 66 sex = 83% F; 17% M age = 73.3 ± N/A BMI = N/A	Primary TKA between March 2010 and November 2013.	PA and SB: Lifecorder EX (Suzuken Co.)	Pre: 1 month Post: 6 months	TKA in older patients led to an increase in the amount of PA.
Matsunaga-Myoji 2020a [52]	Prospective observational study	n = 153 sex = 84.1% F; 15.9% M age = 61.8 ± 7.9 BMI = 23 ± 3.5	Primary TKA between March 2010 and November 2013.	PA and SB: Lifecorder EX (Suzuken Co.)	Pre: 1 month Post: 6 months, 24 months	MVPA and the number of steps was significantly increased 1 year after the operation.
Matsunaga-Myoji 2020b [53]	Prospective observational study	n = 58 sex = 84.5% F; 15.5% M age = 72.6 ± 6 BMI = 26.1 ± 4.4	Primary THA between October 2010 and November 2011.	PA and SB: Lifecorder EX (Suzuken Co.)	Pre: 1 month Post: 6 months, 46 months and 60 months	PA after 6 months exhibited pre-operation levels. Only MVPA increased between 2 and 6 months. Patients can expect PA to continue to improve up to 2 years after TKA.
Moellenbeck 2020a [54]	Prospective observational study	n = 16 sex = 43.8% F; 56.2% M age = 68.9 ± 6.8 BMI = 26.4 ± 4.3	Elective THA.	PA and SB: ActiGraph wGTX3-BT Firmware 1.9.2 (ActiGraph LLC)	Pre: NA Post: 8.9 ± 2.3 months	Sedentary behavior did not show any post-operative change, even though short interruptions of sedentary activity were taken into account.
Moellenbeck 2020b [55]	Prospective observational study	n = 24 sex = 54% F; 46% M age = 69.4 ± 7.2 BMI = 26.6 ± 3.8	Conventional TKA or THA.	PA and SB: ActiGraph wGTX3-BT Firmware 1.9.2 (ActiGraph LLC)	Pre: 2–3 weeks Post: 12.4 ± 1.2 months	The habitual activity of patients stayed the same one year after the operation.
Oka 2020 [56]	Prospective observational study	n = 82 sex = 82% F; 18% M age = 72.1 ± 5.9 BMI = 26.1 ± 3.7	Primary TKA with medial parapatellar approach between June 2016 and June 2019.	PA and SB: Active Style Pro HJA-350IT (Omron Healthcare)	Pre: 1 month Post: 3 months and 6 months	Physical activity level was not increased after operation.
Rezzadeh 2019 [57]	Prospective observational study	n = 18 sex = 50% F; 50% M age = 66.3 ± 9.4 BMI = 28.7 ± 4.5	Unilateral TKA.	PA and SB: Accelerometer (not described)	Pre: 185.8 ± 141.6 days Post: 544.2 ± 141.6 days	No significant difference between post-operative and pre-operative patients.
Thewlis 2019 [58]	Prospective observational study	n = A: 29; B: 4; C: 18 sex = NA age =A: 63 (24–87); B: 69 (64–77); C: 65 (41–83) BMI = A: 30.8 (21.4–40.7); B: 23.6 (22.8–24.4); C: 28.9 (18.6–40.0); n = 58	Primary THA between August 2016 and February 2018.	PA and SB: Wrist worn accelerometer (GeneActiv)	Pre: within 4 weeks Post: 2 weeks, 6 weeks, 12 weeks, 26 weeks	Physical activity did not significantly increase after operation.
Tobinaga 2019 [59]	Prospective observational study	sex = 84.5% F;15.5% M age = 74.6 ± 6.5 BMI = 26.3 ± 4.2	Unilateral TKA.	Performance: TUG	Pre: NA Post: 3 months	Performance was significantly increased after 3 months.
Twiggs 2018 [60]	Prospective observational study	n = 91 sex = 50.5% F; 49.5% M age = 67.5 ± 13.1 BMI = 30.1 ± 6.3	TKA over 21 months period between December 2013 and September 2015.	PA and SB: tri-axial accelerometer FitBit Flex	Pre: 2 weeks Post: 1 day, 6 weeks	The number of steps did not increase after 6 weeks post-operation.
Vissers 2013 [61]	Prospective observational study	n = 44 sex = 59.1% F; 40.9% M age = 63.8 ± 9.4 BMI = 29.7 ± 5	Posterolateral approach THA. TKA procedures using computer navigation (Brainlab, Faldkirchen, Germayn) were performed.	PA and SB: Rotterdam Activity Monitor—AM (Vitaport Technology Temec Instruments)	Pre: 2 months Post: 6 months, 48 months	Performance was significantly increased after the operation.

BMI—body mass index; F—female; M—male; NA—not applicable; TKA—total knee arthroplasty, THA—total hip arthroplasty; PA—physical activity; SB—sedentary behavior; MVPA—moderate to vigorous physical activity; TUG—timed up and go test; 6MWT—6-min walking test.

**Table 2:** Comparison of patients and matched controls in PA, pre-operation and 12 months post-operation.

Time after the Operation	Variable	SMD (95% CI) *	Studies (Participants)	Effect Size	*p*	I^2^	Raw Difference (If Applicable)
Pre-operation	Number of steps	−1.02	4 (344)	Very large	<0.001	62%	−2892.2 steps/day
12 months post-operation	MVPA	−0.97 (−2.39, 0.46)	3 (641)	Very large	0.180	97%	/
	Number of steps	−0.75 (−1.89, 0.38)	3 (372)	Large	0.190	94%	−2671.2 steps/day

* Negative SMD—lower value in patients; SMD—standardized mean difference; I^2^—I-squared statistics for study heterogeneity; MVPA—moderate to vigorous physical activity.

**Table 3:** Comparison of the patients in PA, SB and functional performance pre-operation and at <3 months, 3–6 months, 6–9 months and >12 months post-operation.

Variable	SMD (95% CI) *	Studies (Participants)	Effect Size	*p*-Value	I^2^	Raw Difference (If Applicable)
<3 months post-operation						
Number of steps	−0.06 (−0.36, 0.23)	7 (792)	Small	0.670	77%	−196.7 steps/day
Timed up and go test	0.27 (−1.50, 2.04)	3 (314)	Small	0.770	98%	+1.58 s
6 Minute Walk Test	0.64 (−0.23, 1.51)	4 (478)	Moderate	0.150	91%	+59.9 m
3–6 months post-operation						
Moderate to vigorous physical activity	0.12 (−0.25, 0.48)	3 (301)	Small	0.530	55%	/
Number of steps	0.15 (−0.12, 0.42)	4 (327)	Small	0.390	25%	+373.8 steps/day
6 Minute Walk Test	0.65 (0.48, 0.82)	5 (605)	Large	<0.01	93%	+90.2 m
6–9 months post-operation						
Moderate to vigorous physical activity	0.33 (0.20, 0.46)	9 (923)	Moderate	<0.001	42%	/
Light-intensity physical activity	0.14 (0.06, 0.35)	8 (889)	Small	0.160	49%	/
Sedentary behavior	−0.00 (−0.18, 0.19)	5 (473)	Small	0.790	53%	−3.72 min/day
Number of steps	0.47 (0.18, 0.76)	12 (1418)	Moderate	<0.001	83%	+1064.1 steps/day
Timed-up and go test	−0.61 (−0.94, −0.28)	3 (364)	Large	<0.001	53%	−1.91 s
6 Minute Walk Test	0.62 (0.26, 0.98)	5 (493)	Large	<0.001	73%	+71.84 m
>12 months post-operation						
Moderate to vigorous physical activity	0.70 (0.53, 0.87)	3 (573)	Large	<0.001	0%	
Number of steps	0.52 (0.36, 0.69)	7 (707)	Moderate	<0.001	13%	+1425.3 steps/say

* Positive SMD—higher values post-operation. SMD—standardized mean difference; I^2^—I-squared statistics for study heterogeneity.

## Data Availability

The data presented in this study are available on request from the corresponding author.

## Supplementary Materials

The following are available online at https://www.mdpi.com/article/10.3390/jcm10245885/s1, Table S1: The study quality assessment.
