# Supplementary material for: Objectively Measured Physical Activity, Sedentary Behavior and Functional Performance before and after Lower Limb Joint Arthroplasty: A Systematic Review with Meta-Analysis
Source: J Clin Med. 2021 Dec 15;10(24):5885. doi: 10.3390/jcm10245885 (PMC8709318; doi:10.3390/jcm10245885)
Supplement: Supplementary file 1 [file jcm-10-05885-s001.zip › jcm-1486286-supplementary.pdf]

Table S1. The study quality assessment.

	Aim	Subjects					Assessment								
	**1.1**	**1.2**	**1.3**	**1.4**	**1.5**	**1.6**	**1.7**	**1.8**	**1.9**	**1.10**	**1.11**	**1.12**			
	Clearly Focused Question	Groups Comparability	Participation Rate	Participation Bias	Attrition Rate/Bias	Comparison of Full & Partial Participants	Clearly Defined Outcomes	Blind Assessment	Comparison of Process Measures	Reliability of assessment of measures	Validity & Reliability of Outcome Measures	More than one assessment of prognostic factor	Cofounding	Statistical analysis with CI	Overall quality
Bade 2014 [46]	Y	Y	Y	N/A	N	N	Y	N/A	Y	Y	Y	N/A	Y	N	+
Brandes 2011 [47]	Y	N/A	N/A	N/A	12-40%	Y	Y	N/A	Y	Y	Y	N/A	Y	N	+
Caliskan 2020 [48]	Y	N/A	N/A	N/A	N	N	Y	N/A	Y	Y	Y	N/A	Y	N	+
Casazza 2019 [49]	Y	Y	N/A	Y	N	N	Y	CS	Y	Y	Y	N/A	Y	N	+
Cooper 2017 [50]	Y	Y	Y	N/A	80%	Y	Y	CS	Y	Y	Y	Y	Y	Y	+ +
Daugaard 2018 [51]	Y	Y	N/A	N/A	N/A	N/A	Y	N	N	Y	Y	N/A	Y	Y	+
Dayton 2016 [52]	Y	N/A	N/A	N/A	N	N/A	Y	N/A	Y	Y	Y	Y	Y	N	+
De Groot 2008 [53]	Y	N/A	N/A	N/A	4%	N/A	Y	N/A	Y	Y	Y	Y	Y	N	+
Dominick 2018 [54]	Y	N/A	N/A	N/A	16%	Y	Y	N/A	Y	Y	Y	Y	Y	Y	+ +
Frimpong 2019 [32]	Y	N/A	N/A	N/A	8%; 22; 38%	N/A	Y	N/A	Y	Y	Y	Y	Y	Y	+ +
Frimpong 2020 [55]	Y	N/A	N/A	N/A	33%; 41%	N/A	Y	N/A	Y	Y	Y	N	Y	Y	+

Fujita 2013 [26]	Y	Y	N/A	N/A	39%	N/A	Y	N	Y	Y	Y	N	Y	N	+
Guedes 2011 [29]	Y	Y	N	N/A	N	N/A	Y	N	Y	Y	Y	N/A	Y	N	+
Güler 2019 [31]	Y	Y	N	N/A	7%	CS	Y	N	N/A	Y	Y	N	Y	Y	+
Hawke 2019 [36]	Y	N/A	N/A	N/A	25%; 32%	Y	Y	N/A	Y	Y	Y	CS	Y	N	+
Heiberg 2013 [56]	Y	N/A	N/A	N/A	6%	N/A	Y	N/A	Y	Y	Y	Y	Y	Y	+
Höll 2018 [33]	Y	N/A	N/A	N/A	N	N/A	Y	N/A	Y	Y	Y	N	Y	N	+
Jeldi 2017 [57]	Y	N/A	N/A	N/A	53%	N/A	Y	N/A	Y	Y	Y	CS	Y	N	+
Kahn 2015 [58]	Y	Y	N/A	N/A	N/A	N/A	Y	N/A	Y	Y	Y	N/A	Y	N	+
Ko 2013 [30]	Y	Y	Y	N/A	N/A	N/A	Y	N/A	Y	Y	Y	N/A	Y	Y	++
Kuhn 2013 [59]	Y	N/A	N/A	N/A	67%	N	Y	N/A	Y	CS	Y	Y	Y	N	+
Lin 2013 [60]	Y	N/A	N/A	N/A	N	N/A	Y	N/A	Y	CS	Y	Y	Y	N	+
Lützner 2014 [61]	Y	Y	N	N/A	27%	N/A	Y	N/A	Y	Y	Y	Y	Y	Y	+ +
Lützner 2016 [62]	Y	N/A	N/A	N/A	27%	N	Y	N/A	Y	Y	Y	N	Y	N	+
Matsunaga-Myoji 2019 [27]	Y	Y	N/A	N/A	N	N	Y	N	N/A	Y	Y	N	Y	N	0
Matsunaga-Myoji 2020a [63]	Y	N/A	N/A	N/A	14%; 38%	N/A	Y	N/A	Y	Y	Y	Y	Y	Y	+ +

Matsunaga-Myoji 2020b [64]	Y	N/A	N/A	N/A	24%; 38%	N/A	Y	N/A	Y	Y	Y	Y	Y	Y	+ +
Moellenbeck 2020a [38]	Y	N/A	N/A	N/A	N	N/A	Y	N/A	Y	Y	Y	N	Y	Y	+
Moellenbeck 2020b [28]	Y	Y	N/A	N/A	N	N/A	Y	N/A	Y	Y	Y	N	Y	Y	+
Oka 2020 [35]	Y	N/A	N/A	N/A	33%	N/A	Y	N/A	Y	Y	Y	N	Y	N	+
Rezzadeh 2019 [65]	Y	Y	N/A	N/A	N	N/A	Y	N/A	Y	Y	Y	Y	Y	N	+
Thewlis 2019 [34]	Y	N/A	N/A	N/A	12%; 16%; 20%; 22%	N	Y	N/A	Y	Y	Y	N	Y	N	+
Tobinaga 2019 [37]	Y	N/A	N/A	N/A	N	N/A	Y	N/A	Y	Y	Y	N	Y	N	+
Twiggs 2018 [66]	Y	N/A	N/A	N/A	27%;25%; 25%	N/A	Y	N/A	Y	Y	Y	N	Y	N	+
Vissers 2013 [67]	Y	N/A	N/A	N/A	46%	N/A	Y	N/A	Y	Y	Y	N	Y	N	+
